# Dietary Intake of Total Carbohydrates, Sugar and Sugar-Sweetened Beverages, and Risk of Inflammatory Bowel Disease: A Systematic Review and Meta-Analysis of Prospective Cohort Studies

**DOI:** 10.3389/fnut.2021.707795

**Published:** 2021-10-01

**Authors:** Zeinab Khademi, Alireza Milajerdi, Bagher Larijani, Ahmad Esmaillzadeh

**Affiliations:** ^1^Students' Scientific Research Center, Tehran University of Medical Sciences, Tehran, Iran; ^2^Department of Community Nutrition, School of Nutritional Sciences and Dietetics, Tehran University of Medical Sciences, Tehran, Iran; ^3^Research Center for Biochemistry and Nutrition in Metabolic Diseases, Institute for Basic Sciences, Kashan University of Medical Sciences, Kashan, Iran; ^4^Obesity and Eating Habits Research Center, Endocrinology and Metabolism Molecular-Cellular Sciences Institute, Tehran University of Medical Sciences, Tehran, Iran; ^5^Food Security Research Center, Department of Community Nutrition, School of Nutrition and Food Science, Isfahan University of Medical Sciences, Isfahan, Iran

**Keywords:** diet, carbohydrates, sugar, sugar-sweetened beverages, inflammatory bowel disease

## Abstract

**Objectives:** No earlier study has summarized findings from prospective cohort studies on the association of dietary carbohydrates, sugar, and sugar-sweetened beverages (SSBs) consumption and risk of inflammatory bowel disease (IBD). The current study was done to quantitatively summarize earlier information from prospective cohort studies on the link between dietary carbohydrates, sugar, and SSBs intake with risk of IBD.

**Methods:** Relevant studies published up to June 2021 were searched through PubMed, Medline, SCOPUS, EMBASE, and Google Scholar with the use of relevant keywords. All prospective cohort studies investigating the association of dietary carbohydrates, sugar, and SSBs consumption with risk of IBD were included.

**Results:** Combining 5 effect sizes from 4 cohort studies, no significant association was found between dietary intake of carbohydrates and risk of ulcerative colitis (UC) (RR: 1.22; 95% CI: 0.70–2.14). The same findings were obtained for risk of Crohn's disease (CD) (RR: 1.06; 95% CI: 0.64–1.75) based on 4 studies with 5 effect sizes. A significant positive association was observed between sugar intake and risk of UC (RR: 1.59; 95% CI: 1.15–2.20), as well as CD (RR: 1.90; 95% CI: 1.06–3.41) when 5 effect sizes from 4 cohort studies were combined. The overall effect size, based on 4 estimates, revealed no significant association between SSBs consumption and risk of UC (RR: 1.02; 95% CI: 0.92–1.12) and CD (RR: 1.22; 95% CI: 0.91–1.64).

**Conclusions:** Summarizing earlier studies, sugar intake was found to be associated with increased risk of IBD and its subtypes. Any significant association between dietary intake of carbohydrates and SSBs and risk of IBD and its subtypes was not found.

## Introduction

Inflammatory bowel disease (IBD) is a chronic, progressive inflammatory disorder of the gastrointestinal tract with a peak onset in adolescence and early childhood. It is associated with an enormous burden on health care system (1–3). Although IBD has traditionally been considered as disease of western countries (4, 5), its incidence has recently increased in newly industrialized countries in Asia, South America, and the Middle East (6, 7).

Although the exact pathogenesis of IBD is not fully understood, available evidence suggested that both genetic and environmental factors play a role in IBD development (8). Breastfeeding, antibiotic use, stress, hygiene, appendectomy, and smoking has been associated with development of IBD. Dietary factors have also been postulated to contribute to this condition. Adherence to Western dietary pattern has been reported as a key factor in the growing incidence of IBD (9). It seems that high intake of sugar and refined carbohydrates in this dietary pattern might play a role. Despite several publications on the association between dietary carbohydrates and risk of IBD, findings are still inconsistent (10–14). This is also the case about sources of dietary carbohydrates like sugar and sugar-sweetened beverages. Sugar intake was associated with increased risk of IBD in some (15, 16), but not all (12, 13, 16–21), studies. The same findings were reported for sugar-sweetened beverages (SSBs) (7, 22, 23).

In a previously published meta-analysis in 2017, the investigators focused on total carbohydrates intake and found no significant positive association between dietary carbohydrates intake and risk of ulcerative colitis (UC) incidence. However, a positive association between sugar intake and UC incidence was achieved (24). Zeng et al. reached the same findings for the association between total dietary carbohydrates and sugar intake and risk of Crohn's disease (CD) (25). Concerning sugar-sweetened beverages, a meta-analysis showed that soft drink consumption might be positively associated with risk of UC (26). A more recent meta-analysis showed the same findings for sugar-sweetened beverages consumption and CD incidence (27). However, the findings might be misleading owing to the lack of inclusion of several published studies in the field (10, 11, 23, 28, 29). Furthermore, inclusion of studies done on children might further complicate the findings (30, 31). It should also be kept in mind that in the previous meta-analyses, case-control studies were combined with prospective cohort studies that might affect the results. The current study was, therefore, done to quantitatively summarize earlier information from prospective cohort studies on the link between dietary carbohydrates, sugar, and sugar-sweetened beverages intake with risk of IBD.

## Materials and Methods

### Search Strategy

Literature databases including PubMed, MEDLINE, SCOPUS, EMBASE, and Google Scholar were searched until June 2021. The following keywords including those from the medical subject headings (MESH) database and non-MESH keywords, were used in this search: (“sucrose” or “sugar” or “carbohydrate” or “sugar-sweetened beverage” or “soft drink”) and (“inflammatory bowel disease” or “Crohn disease” or “ulcerative colitis”). No language or time limitations were performed. In addition, references from the extracted articles and reviews were also reviewed to avoid missing any publication. Unpublished studies, congress abstracts, dissertations, and patents were not included in this meta-analysis. Duplicate citations were removed. This meta-analysis has been conducted based on the Preferred Reporting Items for Systematic Reviews and Meta-analyses (PRISMA) guidelines (32).

### Inclusion Criteria

All prospective cohort or nested case-control studies that reported hazard ratios (HRs) or relative risks (RRs) or odds ratio (OR) and 95% Confidence Intervals (CIs) for IBD across categories of dietary carbohydrates, sugar, and sugar-sweetened beverages intake was included. If several publications were reported based on the same dataset, only the most complete one was included. In addition, if a study had reported data for specific subgroups, results for the whole population were included.

### Exclusion Criteria

**Figure 1:**
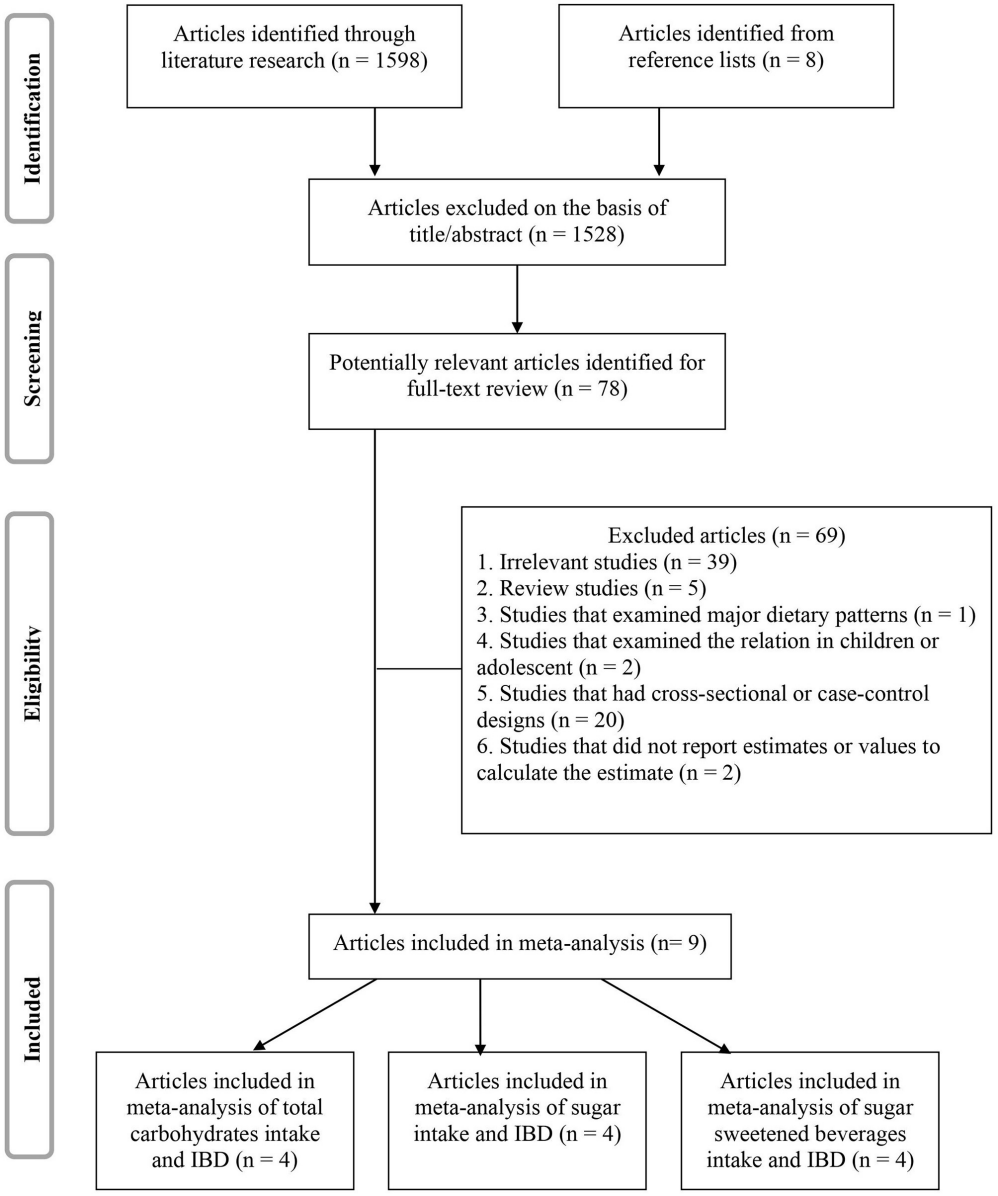
Flow diagram of study selection.

Studies were excluded if they: (1) were done on animals, pregnant women, or children, (2) had cross-sectional or case-control designs or were clinical trials, (3) had examined dietary patterns, and (4) had reported only *p*-values and did not provide any measures enabling us to calculate the effect size for the association. After these exclusions, nine papers remained for systematic review in the present study. Flow-diagram of study selection is shown in Figure 1.

### Data Extraction

From each eligible study, two independent reviewers extracted the following information: first author's name, publication year, country, age range of study participants, participants' sex, sample size, number of cases with IBD, follow-up duration, type of exposure, methods used to assess exposure, methods used to assess outcome, relevant effect sizes (OR, HR, RR) with 95% CIs for IBD, and the covariates that were adjusted for.

### Quality Assessment of Studies

The Newcastle-Ottawa Scale (NOS) (33) adapted for cohort and case-control studies was used to examine methodological quality of the included studies. The Newcastle-Ottawa Scale considers the selection of study groups (0–4 scores), adequacy of adjustment for confounding (0–2 scores), and assessment of the outcome of interest (0–3 scores). The maximum score of 9 can be assigned to each study. In the present study, publications with at least seven scores were considered as high-quality studies, and those with a score of 3–6 were considered moderate-quality. Any discrepancies were resolved by discussion.

### Statistical Analysis

To compute overall effect size, the log HRs, RRs or ORs and its standard error were calculated based on reported HRs, RRs, or ORs, and their 95% CIs. The pooled effect size was calculated using random-effects model, which takes between-study variation into account. Between-study heterogeneity was examined using Cochran's *Q*-test and *I*^2^ statistic. Subgroup analysis was not performed due to the limited numbers of included articles. All statistical analyses were conducted using STATA version 19.0 software (Stata Corp LP, College Station, TX). *P* < 0.05 were considered statistically as significant.

## Results

### Findings From Systematic Review

The characteristics of nine studies included in this systematic review are presented in Table 1. These studies were published between 1992 and 2019. Among included studies, five used a prospective cohort design (10, 16, 23, 28, 29), whereas the four remaining studies were nested case-control studies (11, 12, 22, 34). Overall, seven publications were reported from European countries (10–12, 16, 22, 23, 29). Others were from US (28), and Asia-pacific region (34). Two studies were conducted on women only (10, 28) and others on both genders. Participants aged between 10 (11) and 83 (23) years. Sample sizes ranged from 227 people in nested case-control studies (29) to 401326 in cohort studies (16).

**Table 1:** General characteristics of included studies.

**First Author (Year)**	**Country**	**Age range**	**Sex**	**Sample size**	**Cases**	**Study design**	**Follow-up duration (y)**	**Exposure assessment method**	**Outcome assessment method**	**Exposure/comparison**	**OR or RR (95%CI)**		Adjustments^*^
Persson et al., 1992 (12)	Sweden	15–79	F/M	755	365	Nested case-control	5	FFQ (40 food categories)	Medical record	Carbohydrates/≥280 vs. ≤209	UC	M: 2.1 (0.5, 8.1) F: 3.7 (0.5, 24.9)	1, 2, 3
											CD	M: 4.0 (0.9, 16.7) F: 1.0 (0.2, 4.3)	
										Sucrose/≥55 vs. ≤29	UC	M: 1.2 (0.5, 3.1) F: 1.9 (0.7, 4.9)	
											CD	M: 1.8 (0.6, 5.0) F: 3.6 (1.4, 8.9)	
										soft drinks/Daily vs. less frequency	CD	F/M: 2.8 (1.6, 4.9)	
Russel et al., 1998 (22)	Netherlands	21–54	F/M	1,304	688	Nested case-control	NR	Specifically designed questionnaire	The criteria of Truelove and Witts	Cola drinks/More than once a week vs. non or once a week	UC	1.2 (0.8, 1.6)	1, 2, 4, 5, 6
									The criteria of Lennard- jones		CD	1.4 (1.0, 2.0)	
Halfvarson et al., 2006 (29)	Sweden, Denmark	NR	F/M	317	227	Cohort	NR	Specifically designed questionnaire	Self-report, medical records confirmed by physician	Additional sugar on porridge/Yes vs. No	UC	2.4 (1.2, 4.9)	7
											CD	1.2 (0.6, 2.6)	
Jantchou et al., 2010 (10)	France	40–65	F	705,445	77	Cohort	10.4	FFQ (208 items/valid)	Clinical, radiological, endoscopic, and histological	Carbohydrates/T3 vs. T1	UC	0.51 (0.24, 1.08)	3, 4, 5, 8, 9, 10, 11, 12
											CD	1.31 (0.42, 4.14)	
											IBD	0.68 (0.37, 1.27)	
Hansen et al., 2011 (11)	Denmark	10–95	F/M	534	267	Nested case-control	NR	Questionnaire proposed by the international organization of IBD	NR	High sugar intake/cases vs. controls	UC	1.68 (0.96, 2.97)	5, 11, 13, 14, 15, 16, 17, 18
											CD	3.50 (1.73, 7.07)	
Chan et al., 2014 (16)	8 European countries	20–80	F/M	401,326	354	Cohort	NR	FFQ (200 items/valid)	Registries	Carbohydrates/Q4 vs. Q1	UC	1.46 (0.62, 3.46)	3, 4, 8
											CD	0.87 (0.24, 3.12)	
										Sugar/Q4 vs. Q1	UC	1.12 (0.57, 2.17)	
											CD	0.76 (0.28, 2.08)	
Ananthakrishnan et al., 2015 (28)	USA	25–42	F	39,511	173	Cohort	19.31	FFQ (124 item/valid)	Self-report, medical records confirmed by physician	Total carbohydrates/Q4 vs. Q1	UC	1.38 (0.8, 2.38)	4, 8, 10, 11, 12, 15, 19, 20, 21
											CD	0.77 (0.40, 1.48)	
Ng et al., 2015 (34)	9 countries/ regions in Asia-Pacific	25–50	F/M	1,382	442	Nested case-control	NR	Questionnaire proposed by International Organization of IBD	Clinical symptoms, endoscopy, histology, and radiology	Soft drinks/≥twice/week	UC	1.55 (0.83, 2.89)	1, 2, 22
											CD	0.75 (0.38, 1.49)	
Khalili et al., 2019 (23), (SMC)	Sweden	44–83	F	38,046	311	Cohort	11	FFQ (valid)	Registries	Sweetened beverages/per 1 daily serving of a sweetened beverage	UC	0.92 (0.74, 1.13)	1, 3, 4, 8, 15, 20, 23, 24
											CD	1.05 (0.79, 1.41)	
Khalili et al., 2019 (23), (CoMS)	Sweden	44–83	M	44,996	181	Cohort	11	FFQ (valid)	Registries	Sweetened beverages/per 1 daily serving of a sweetened beverage	UC	1.01 (0.94, 1.08)	1, 3, 4, 8, 15, 20, 23, 24
											CD	0.99 (0.87,1.12)	

*Y, year; OR, odds ratio; RR, Relative risks; CI, confidence intervals, F, female; M, male*.

* *1, age; 2, sex; 3, total energy; 4, smoking; 5, educational level; 6, nutritional factor; 7, Multiple testing within each of the groups. 8, BMI; 9, alcohol; 10, physical activity; 11, oral contraceptive intake; 12, menopause hormonal treatment;13, appendectomy; 14, tonsillectomy; 15, fiber consumption; 16, sugar consumption; 17, coffee consumption; 18, eggs consumption;19, menopausal status; 20, non-steroidal anti-inflammatory drug use; 21, vitamin D intake; 22, country income; 23, cohort; 24,total protein intake*.

Total carbohydrates, sugar, and sugar-sweetened beverages were considered as exposure in the included studies. Four studies considered total carbohydrates (10, 12, 16, 28) intake as the exposure. Out of four studies that assessed sugar intake (11, 12, 16, 29), one study had considered total sugar (16), one had assessed sucrose (12), one had considered high sugar on porridge intake (11), and the last one had considered additional sugar intake (29). Studies that assessed sugar-sweetened beverages, had considered consumption of cola drinks (22) and soft drinks (12, 23, 34). Most included studies had used food frequency questionnaire (FFQ) (10, 12, 16, 23, 28) to assess dietary intakes, others used specifically designed questionnaires (22, 29) and questionnaires proposed by the International Organization of IBD (11, 34).

The outcome of interest in these studies were UC (11, 12, 16, 22, 23, 28, 29, 34), CD (11, 12, 16, 22, 23, 28, 29, 34), and IBD (both UC and CD) (10), which were assessed through participants' self-reports and medical records confirmed by a physician (28, 29), registries (16, 23), only medical records (12), criteria of Truelove and Witts criteria of Lennard-jones (22), and by the clinical examinations (10, 34). Only one study did not report assessment tool (11). Most studies had reported OR (11, 16, 22, 29, 34) or RR/HR (10, 12, 23, 28). Adjustment for dietary intakes was performed in most studies (10–12, 16, 22, 23, 28).

### Findings for the Association Between Total Carbohydrates Intake and Risk of IBD

**Figure 2:**
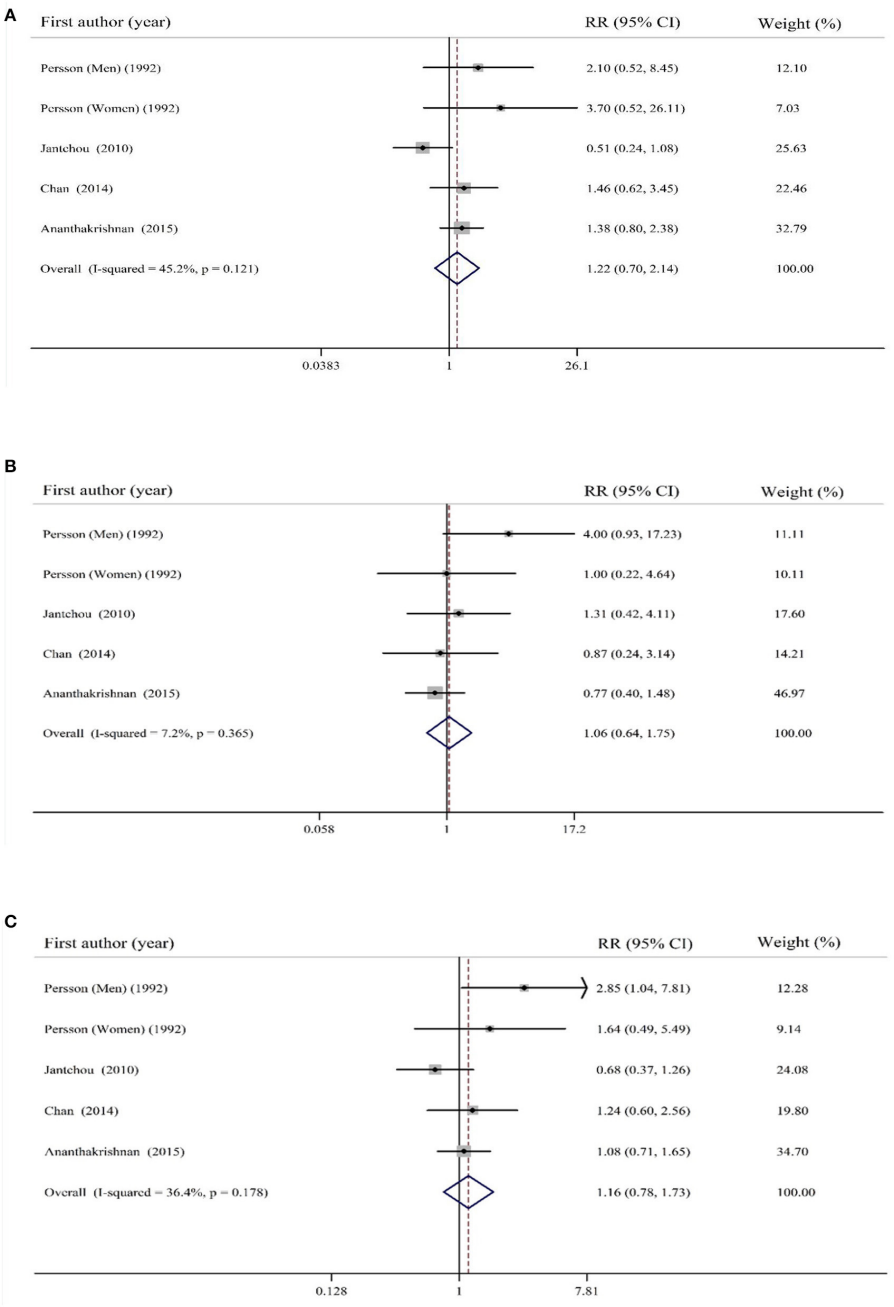
Forest plot for the association of total carbohydrates intake with risk of **(A)** ulcerative colitis (UC), **(B)** Crohn's disease (CD), and **(C)** total inflammatory bowel disease (IBD). Diamonds represent pooled estimates from random-effects analysis. Horizontal lines represent 95% CIs.

Combining five effect sizes from four cohort studies, no significant association was found between dietary intake of carbohydrates and risk of UC (RR: 1.22; 95% CI: 0.70–2.14, *I*^2^= 45.2%, *p* = 0.12; Figure 2A). The same findings were obtained for the association between dietary intake of carbohydrates and risk of CD when we combined five effect sizes from four studies (RR: 1.06; 95% CI: 0.64–1.75, *I*^2^ = 7.2%, *p* = 0.36; Figure 2B). By pooling studies on UC and CD, no significant association was found between total dietary carbohydrates intake and risk of total IBD (RR: 1.16; 95% CI: 0.78–1.73, *I*^2^ = 36.4%, *p* = 0.17; Figure 2C).

### Findings for the Association Between Sugar Intake and Risk of IBD

**Figure 3:**
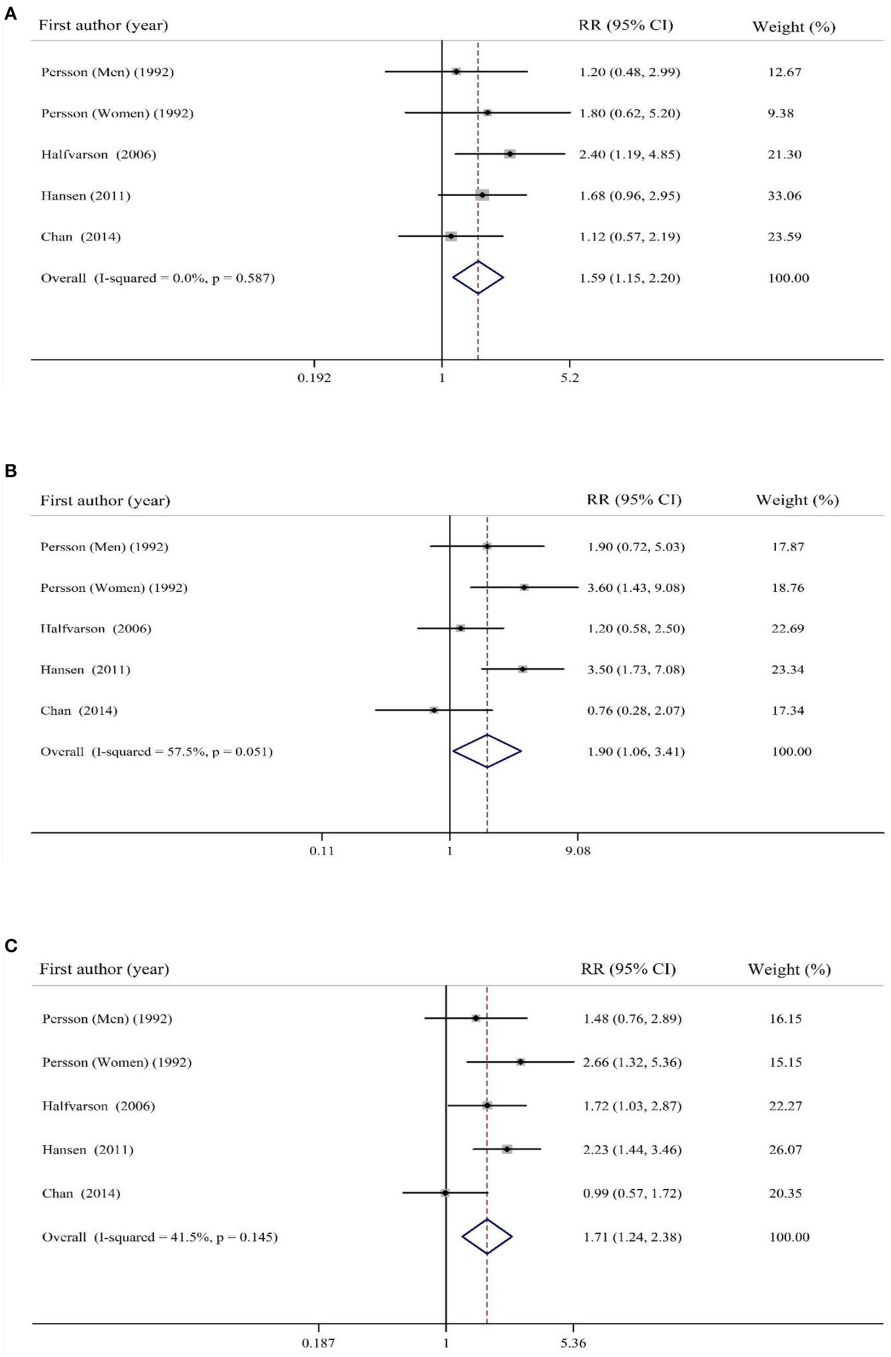
Forest plot for the association of sugar intake with risk of **(A)** UC, **(B)** CD, and **(C)** total IBD. Diamonds represent pooled estimates from random-effects analysis. Horizontal lines represent 95% CIs.

Combining five effect sizes from four cohort studies, a significant positive association was observed between dietary intake of sugar and risk of UC (RR: 1.59; 95% CI: 1.15–2.20, *I*^2^ = 0%, *p* = 0.58; Figure 3A). In addition, pooling five effect sizes provided by four cohort studies, a significant positive association was observed between sugar intake and risk of CD (RR: 1.90; 95% CI: 1.06–3.41, *I*^2^ = 57.5%, *p* = 0.051; Figure 3B). When studies on UC and CD were combined, a significant positive association was, again, found between sugar intake and risk of IBD (RR: 1.71; 95% CI: 1.24–2.38, *I*^2^ = 41.5%, *p* = 0.14; Figure 3C).

### Findings for the Association Between Sugar-Sweetened Beverages Consumption and Risk of IBD

**Figure 4:**
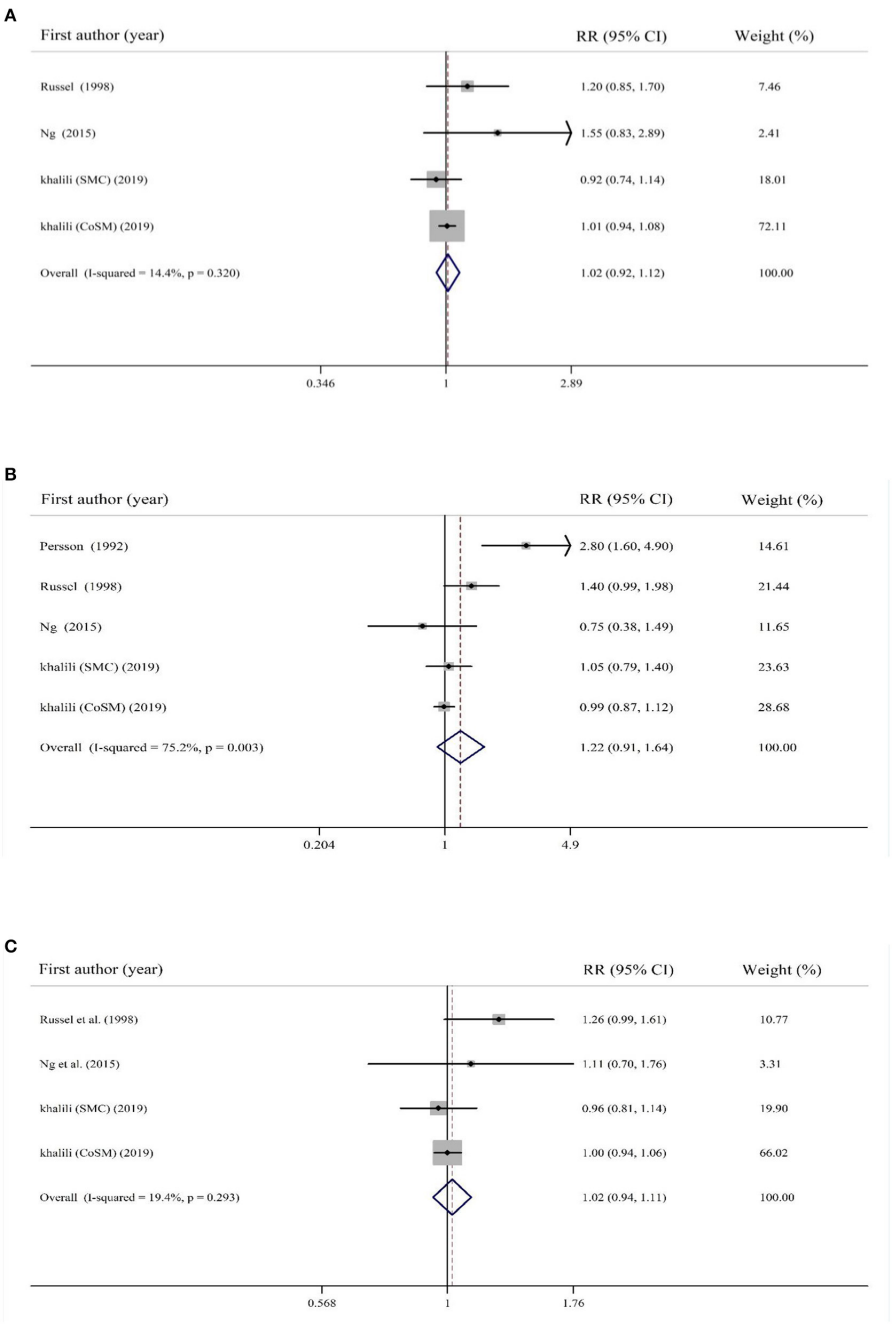
Forest plot for the association of sugar-sweetened beverages consumption with risk of **(A)** UC, **(B)** CD, and **(C)** total IBD. Diamonds represent pooled estimates from random-effects analysis. Horizontal lines represent 95% CIs.

Overall effect size based on four estimates revealed no significant association between SSBs consumption and risk of UC (RR: 1.02; 95% CI: 0.92–1.12, *I*^2^ = 14.4%, *p* = 0.32; Figure 4A). Additionally, the same findings were obtained for risk of CD (RR: 1.22; 95% CI: 0.91–1.64, *I*^2^ = 75.2%, *P* = 0.003; Figure 4B). Combining studies on both UC and CD, no significant association was observed between SSBs consumption and risk of total IBD (RR: 1.02; 95% CI: 0.94–1.11, *I*^2^ = 19.4%, *p* = 0.29; Figure 4C).

## Discussion

In this meta-analysis on nine studies, any significant association was not found between total carbohydrates intake as well as SSBs consumption and risk of IBD. However, high consumption of sugar was significantly associated with increased risk of IBD.

The IBD incidence has increased worldwide. It usually occurs in adolescents and has a relapsing course, therefor can cause enormous socioeconomic burden and reduction of quality of life for the patients (2, 4). Our findings suggested lack of any significant association between total dietary carbohydrates and risk of IBD. These findings were in line with a previous meta-analysis (35). However, the findings of previous meta-analysis might be misleading, due to lack of considering a published study in the field (28), inclusion of a study done on children (30), and two publications that were reported based on the same dataset (16, 36). Our findings were also in agreement with the results of another meta-analysis on the association between macronutrients intake and risk of CD (25). However, they did not consider two published studies in the field (10, 28). In another meta-analysis on dietary carbohydrates intake and risk of UC (24), in which no association was found, the authors did not consider a published study in the field (28) and included findings from two publications with the same population (16, 36). Overall, it seems that there is no significant association between dietary carbohydrates intake and risk of IBD. However, as studies in this field are limited, further studies with large sample sizes are required to come to a definite conclusion.

To the best of our knowledge, this is the first comprehensive meta-analysis on the association between sugar consumption and risk of IBD. In a previous meta-analysis, Wang et al. found no association between sugar consumption and risk of UC based on two studies. However, they reached a positive significant association between sucrose intake and risk of UC (24), although they did not consider all available published studies (11, 16, 29). In a meta-analysis on dietary intakes of monosaccharides, disaccharides, and starch in relation to risk of CD, the investigators found a marginally significant association between dietary sucrose intake and CD risk (25). Two relevant studies (11, 29) were not included in their analysis as well. Overall, it seems that sugar consumption might be associated with increased risk of UC, CD, and total IBD. However, additional cohort studies in this regard might further help to shed light on this issue.

In the current meta-analysis, any significant association was not found between SSBs consumption and risk of IBD. This was in opposite to previous meta-analyses (26, 27). In an earlier meta-analysis based on five studies (27), consumption of SSBs was positively associated with risk of UC. However, the investigators in that meta-analysis included a study that was done on children (31). In addition, they missed some other relevant publications (23). In another meta-analysis, Nie et al. (26) found that high soft drink intake was associated with an increased CD risk. That meta-analysis had also the same problems as mentioned above (22, 31). Overall, based on available studies, one can conclude that despite the detrimental effects of SSBs consumption on human health, it is a bit early to conclude that their consumption might increase the risk of IBD. Additional studies are required in this field to come to a definite conclusion.

A significant association was not found between total carbohydrates intake and risk of IBD. This might be explained by the inclusion of non-digestible carbohydrates, including dietary fiber, in the calculation of total carbohydrates intake in the included studies. Non-digestible carbohydrates cannot be hydrolyzed in the small intestine, but are fermented by the gut microbiota and enhance the production of short-chain fatty acids (SCFAs), which can, in turn, regulate gut immune and barrier function and suppress inflammation through suppression of NF-kB (37–41).

Total carbohydrates intake was considered as one of the exposures in the current study. However, it seems that type of dietary carbohydrate is more important in determining the risk of IBD than total amount of carbohydrates. In this study, it was found that high sugar intake was associated with an increased risk of IBD. Experimental studies have indicated that consumption of a high-sugar diet promotes intestinal dysbiosis, the expansion of bacterial pathobionts, and inflammation. Exposure to a high sugar diet increases susceptibility to colitis by reducing SCFA production and increasing gut permeability. It is also noted that high levels of luminal sucrose might have direct effects on loss of intestinal barrier function (39, 41, 42). Additionally, high consumption of simple carbohydrates is mostly associated with high intake of total energy and subsequently a higher risk of overweight and obesity (43), which can, in turn, increase the risk of inflammatory states (44). With regard to SSBs, a significant association was not found. The association between SSBs consumption and IBD risk might be neutralized by benefits from other food group intake. In a previous prospective cohort study, sugar and soft drinks pattern was linked with UC risk, only if participants had low vegetable intake (45). In addition, despite the role of SSBs consumption in several chronic conditions, they have not been associated with inflammatory markers including serum C-reactive protein (CRP) and Interleukin-6 (IL-6) in some studies (46, 47). Given the inflammatory nature of IBD, it seems that additional studies are required to shed light on the relation between SSBs consumption and risk of IBD.

Along with several strengths, this study has some limitations as well. The analysis was confined to prospective cohort studies because findings from case-control and cross-sectional studies are subject to bias. Using the estimates with a maximum adjustment was another strength of the study. One limitation of the current meta-analysis is that the included studies have used different dietary assessment tools to examine total carbohydrates, sugar, and SSBs intake. Despite the use of FFQ in most studies, it must be kept in mind that dietary data from FFQ might be overestimated. Additionally, misclassification of study participants in terms of dietary intakes is always a problem in these studies. Moreover, most studies had considered baseline dietary intake of participants as the main exposure rather than the average of repeated assessments. Included studies had used different diagnostic criteria for defining IBD. The inconsistent adjustment for potential confounders among included studies might also contribute to between-study heterogeneity. The OR, HR, or RR was extracted with the maximum adjustment for potential confounders. However, the extent to which these estimates were adjusted and the residual confounding by other unmeasured factors should be considered.

In conclusion, summarizing earlier studies, it was found that sugar intake was associated with increased risk of IBD and its subtypes. Any significant association between dietary intake of carbohydrates and SSBs, and risk of IBD and its subtypes was not found. Further studies, especially of prospective design from developing countries, are needed to expand the current knowledge in this regard.

## Data Availability Statement

The original contributions presented in the study are included in the article/supplementary material, further inquiries can be directed to the corresponding author/s.

## Author Contributions

ZK and AM contributed to design, search, statistical analyses, and manuscript drafting. AM and BL contributed to design and statistical analyses. AE supervised the study. All authors approved the final manuscript for submission.

## Funding

This study was supported by Research Council of School of Nutritional Sciences and Dietetics, Tehran University of Medical Sciences, Tehran, Iran.

## Conflict of Interest

The authors declare that the research was conducted in the absence of any commercial or financial relationships that could be construed as a potential conflict of interest.

## Publisher's Note

All claims expressed in this article are solely those of the authors and do not necessarily represent those of their affiliated organizations, or those of the publisher, the editors and the reviewers. Any product that may be evaluated in this article, or claim that may be made by its manufacturer, is not guaranteed or endorsed by the publisher.
